# Reproductive functions in *Desmodus rotundus*: A comparison between seasons in a morphological context

**DOI:** 10.1371/journal.pone.0205023

**Published:** 2018-10-17

**Authors:** Ana Cláudia Ferreira Souza, Felipe Couto Santos, Daniel Silva Sena Bastos, Marcela Nascimento Sertorio, João Paulo Gusmão Teixeira, Kenner Morais Fernandes, Mariana Machado-Neves

**Affiliations:** 1 Department of General Biology, Federal University of Viçosa, Viçosa, Minas Gerais, Brazil; 2 Department of Animal Science, Federal University of Viçosa, Viçosa, Minas Gerais, Brazil; 3 Department of Animal Biology, Federal University of Viçosa, Viçosa, Minas Gerais, Brazil; 4 Department of Entomology, Federal University of Viçosa, Viçosa, Minas Gerais, Brazil; Faculty of Animal Sciences and Food Engineering, University of São Paulo, BRAZIL

## Abstract

Reproductive seasonality in Neotropical bats has been assessed to the better understand their reproductive behavior. This knowledge is especially important for the control of *Desmodus rotundus* population as it is a transmitter of rabies virus. Therefore, we aimed to evaluate the functional activity of testis and epididymis of *D*. *rotundus* in dry and rainy seasons under a morphological approach. We observed an increase in tubular diameter and epithelial height of the seminiferous tubules during the rainy season. In the latter, additionally, stereological analysis of the testis showed increased proportion of seminiferous epithelium and reduced percentage of lumen. The sperm number in caput/corpus epididymis increased in rainy season, whereas sperm count and transit time were reduced in cauda region. These alterations were probably related to the recovery of epithelium activities after mating season in dry season. Despite altered nuclear and cytoplasm parameters of Leydig cells between seasons, the volume and number of these cells were constant. Moreover, no change in serum testosterone levels, daily sperm production, and apoptotic index were observed, which indicates that the reproductive pattern in *D*. *rotundus* does not change between seasons. Our study offers a baseline for the management of vampire bat population as an attempt to control rabies disease.

## Introduction

Bat species inhabiting seasonal environments usually pattern their mating season to precede or coincide with periods when weather conditions and food availability are favorable for successful reproduction [1,2]. On the other hand, there are bats in which the reproductive pattern is not restricted to seasonal variations [3,4]. In males, reproductive strategies may be relied on individual body condition, food availability, expression of hormonal receptors (e.g., androgen receptor, estrogen receptor, LH receptor and FSH receptor) or testicular recrudescence [5–7]. Nevertheless, their sexual activity is strongly associated with female cycles that can be monoestrous or polyestrous, and non-seasonal or seasonal [1].

Over the last decades, reproductive seasonality in Neotropical bats has been assessed under ecological, behavioral, and morphological approaches [8–11]. Among them, the latter is considered more precise because involves the evaluation of morphological and functional features of reproductive organs. Several bat species have been identified as seasonal and non-seasonal breeders using histological and morphometric methods [4,6,12,13]. The histological quantification of the testis is a basic requirement for studies involving reproductive variables [14]. The use of morphometric and stereological approaches in the testicular compartments allows evaluating the activity of the organ and its relationship with the seasonal reproduction period of the animals [7,15]. Particularly, the quantification of Leydig cells, which are responsible for testosterone production, allows for making inferences about the spermatogenesis and gonadal dynamics [16]. Additionally, the presence of spermatozoa in testis lumen indicates sexual maturity of the animals [3,17].

The common vampire bat *Desmodus rotundus* is a phyllostomid widely distributed in Brazil. This species is one of the three bats species that feeds exclusively on blood, mainly from cattle, pigs, and horses. For that reason, vampire bats are often found inhabiting caves near domestic livestock farms [18,19]. Consequently, *D*. *rotundus* is considered one of the most important vectors of rabies virus, and many deaths have been associated with its bites [20,21]. Thereby, the better understanding of the reproductive physiology in *D*. *rotundus* is important for the development of alternative methods to control its population.

It is known that *D*. *rotundus* lives in colonies with a dominant male forming a harem [22]. Although the females are characterized as polyestrous, studies have shown that these animals present reproductive seasonality, with peak of births and lactation in spring-summer (rainy season) [23–27]. According to Lord [25], the seasonal mating of the common vampire bats results in an influx of young bats into the bat populations, which coincides with the increase in bovine rabies during the rainy season.

Despite the information available on the reproductive behavior of this species, little is known about its male reproductive physiology. Some studies have focused on its spermatogenesis [28] and structural features of the testis and epididymis [29–31]. Nevertheless, no studies were found evaluating seasonal influences on male reproductive parameters of these bats. In the present study, we evaluated the functional activity of testis and epididymis of *D*. *rotundus* in dry and rainy seasons under a morphological approach. For that end, we focused on testicular stereology and apoptotic index, serum testosterone concentration, and epididymal function in animals captured during both seasons.

## Materials and methods

### Study area, animals and tissue collection

Adult male bats (*D*. *rotundus*) were captured at night using mist nets, positioned around a cave in Piau, Minas Gerais State, Brazil (21°27’09.1”S and 43°14’50.6”W; 475 m altitude). This is a mountainous region localized in the Atlantic Forest biome, with a humid subtropical climate (Cwa) of Köopen (rainy summers and dry winters). The means of temperature, rainfall, photoperiod, and relative humidity during the period of captures were 17.3°C, 29.30 mm, 10.98 time/light/day and 81.2% in the dry season (April-September), respectively, and 21.6°C, 122.30 mm, 12.59 time/light/day and 76% in the rainy season (October to March), respectively (National Institute of Meteorology - http://www.inmet.gov.br/portal/).

The animal sampling was performed in the end of April (*n =* 5), which marks the beginning of the dry season, and June (Dry season; *n =* 5), whereas other 10 males were captured at the end of October, when starts the rainy season. All captures occurred during 2016 in nights without a full moon. The animals were identified according to the identification key for Brazilian bats [32]. To ensure adult status, only animals with complete ossification of the cartilaginous epiphyseal growth plates of the fourth metacarpal phalangeal joint were selected [33].

After the captures, the animals were transported to the Laboratory of Structural Biology at the Federal University of Viçosa (UFV), where they were placed in individual steel cages protected from light, at room temperature, and with free access to water, only during the period between the end of the captures and the next morning. This short period in captivity was determined for acclimatization of the animals as an attempt to minimize stress conditions and possible alteration in serum cortisol levels [34,35]. Then, bats were weighted and killed by intraperitoneal injection of sodium pentobarbital (40 mg/Kg/body weight), followed by guillotining [30]. The blood flowing from the trunk was immediately collected for hormonal analysis. The testes and epididymides were removed, dissected, and weighted. The gonadosomatic index (GSI), which indicates the percentage of body mass allocated on the testes, was obtained by computing the ratio between gonadal weight (GW) and body weight (BW), where GSI = GW/BW x 100 [36]. While right testes were used for morphological, stereological and immunofluorescence analyses, left testes and epididymides were used for sperm counts, daily sperm production and sperm transit time evaluation.

The fieldwork was performed in cooperation with Instituto Mineiro de Agricultura (IMA; State Institute of Agriculture) responsible for the control of *D*. *rotundus* population in Minas Gerais state, Brazil, according to the occurrences of bat bites in livestock informed by farmers to this Institute. Therefore, the owners of the private lands gave us permission to conduct the study on their lands. Bats capture was authorized by the Chico Mendes Institute for Biodiversity Conservation (ICMBio, license number 40629–1 to M. Machado-Neves). All experimental procedures were conducted in accordance with the recommendations of the Guide for the Care and Use of Laboratory Animals of the National Institutes of Health, and were approved by the Ethics Committee in Animal Use of UFV, Brazil (CEUA process number 59/2014). *D*. *rotundus* is a wild species protected by federal laws in Brazil (Brazilian Institute of Environment–IBAMA). However, it is not listed as endangered species on the International Union of Conservation of Nature (IUCN) Red List of Threatened Species. Moreover, its populations are controlled in regions with rabies cases incidence, once this species is the most important vector of rabies virus [20,21]. Thus, the number of animals used in this study was the maximum obtained from a balance between the minimum number necessary for statistical analyses and the maximum number of animals that we could capture in cooperation with IMA, without disrupting colonies and the population control of the species carried out by the Institute.

### Histology and histomorphometry

The right testes (*n =* 5/group) were immersed in Karnovsky fixative solution (2.5% glutaraldehyde, 4% paraformaldehyde in 0.1 M pH 7.2 sodium phosphate buffer) [37] for 24 h. Then, testicular fragments were dehydrated in crescent ethanol series, and embedded in 2-hydroxyethyl methacrylate (Historesin; Leica Microsystems, Nussloch, Germany). Tissue sections at 3 μm thickness were obtained using a rotary microtome (RM 2255; Leica Biosystems, Nussloch, Germany), stained with toluidine blue-sodium borate (1%), and analyzed using light microscope (Olympus CX40; Tokyo, Japan).

Morphometric and stereological analysis of the testes were performed using the Image-Pro Plus 4.5 (Media Cybernetics, Silver Spring, USA) software. For this purpose, digital images of testicular parenchyma were obtained using a light microscope (Olympus BX-53; Tokyo, Japan) equipped with a digital camera (Olympus DP73; Tokyo, Japan). The volumetric rates between tubular and intertubular compartments of the testes were obtained by counting 2,660 points projected onto 10 images captured in histological slides per animal. Coincident points were recorded in seminiferous tubules (tunica propria, epithelium and lumen) and intertubule. The percentage of each testicular component was calculated according to Souza et al. [38]. Considering the density of the testis as 1, the testicular weight was considered the same as its volume [14]. These data were used to calculate the volume of tubule and intertubule compartments. The tubule-somatic index was obtained by dividing the seminiferous tubule volume by the body mass and multiplying the result by 100 [36]. The diameter of the seminiferous tubule and the height of the seminiferous epithelium were obtained by randomly measuring 30 tubular cross-sections, regardless the stage of the epithelium within the cycle [14]. The epithelium height of each tubule was measured from the tunica propria to the tubular lumen and represented as the average of two diametrically opposed measurements. The total length of the seminiferous tubules per testis and per gram of testes was estimated according to Souza et al. [38].

To analyze the intertubular elements (lymphatic space, connective tissue, blood vessels, macrophages and Leydig cells), 1,000 intersections were counted on images at 400x magnification, using a grid with 266 intersections. The proportion and volume of each intertubular component was obtained according to Souza et al. [38]. The diameter of Leydig cell nucleus was measured in 30 cells per animal and the nuclear volume was calculated using the following formula: 4/3πR^3^ (R = nuclear diameter/2). The cytoplasm volume was estimated by multiplying the volumetric proportion of cytoplasm by the nuclear volume, divided by the nuclear proportion. Adding these values, the individual Leydig cell volume was determined. The number of Leydig cells per testis was estimated dividing the total Leydig cell volume per individual Leydig cell volume. This value was then divided by the testes weight, to estimate the number of Leydig cell per gram of testis. The leydigosomatic index, which quantifies the investment in Leydig cell related to body weight, was estimated relating Leydig cell volume in the testicular parenchyma by the body weight [39].

### Immunofluorescence

After fixation in 4% paraformaldehyde, fragments of right testes (*n =* 5/group) were washed three times, for 30 min each, in sodium phosphate buffer 0.1 M at pH 7.2 containing 1% of Triton X-100 (PBST). Tissues were then incubated with cleaved-caspase 3 primary antibody (cat #9661; Cell signaling Technology, Inc.) diluted at 1:200 in PBST for 24 h at 4°C. The samples were once again washed three times in PBS for 5 min each and incubated with FITC-conjugated anti-rabbit IgG secondary antibody (cat #F0382; Sigma-Aldrich) diluted at 1:500 in PBST for 24 h at 4°C in the darkness. After three washes in PBS, testicular fragments were embedded in 2-hydroxyethyl methacrylate (Historesin; Leica Microsystems) and tissue sections with a thickness of 10 μm were obtained using a rotary microtome (RM 2255; Leica Biosystems). The slides were stained with DAPI for 30 min, washed three times in PBS for 5 min each and mounted in 50% sucrose. All the procedures were made protected from light. Apoptotic cells were counted in 10 histological images captured randomly in histological slides per animal at 200x magnification, using an inverted fluorescence microscope (EVOS FL; Advanced Microscopy Group, Bothell, WA, USA). The area of the images was measured using Image-Pro Plus 4.5 (Media Cybernetics, Silver Spring, USA) software. Each histological section showed an area of 0.238 mm^2^, obtaining a total area of 2.38 mm^2^. The number of apoptotic cells per unit histological area was calculated according to the relation QA = Σ apoptotic cells/total area [40].

### Determination of serum testosterone

Blood samples collected during euthanasia (*n =* 5/group) were centrifuged at 2,000*g* for 15 min, and the serum was stored at -20°C. Quantification of serum testosterone was assessed using a species independent Testosterone ELISA kit (ADI-900- 065; Enzo Life Sciences, Plymouth, PA, USA). This is a competitive enzyme immunoassay kit that uses a monoclonal antibody to testosterone to bind the hormone itself or another alkaline phosphatase molecule that has testosterone covalently attached to it. The assay was performed in accordance with the manufacturer`s instructions. In brief, standards (n = 5) of known testosterone concentration (7.81–2,000 pg/ml) were prepared following the protocol instruction using PBS plus bovine serum albumin (BSA). Then, the latter was established as zero standard. Serum samples were evaluated undiluted. For samples with testosterone concentration above the detection limit of the assay, we diluted the serum using PBS + BSA to obtain a reliable testosterone concentration on a subsequent assay. The sensitive range of the assay was 7.81–2,000 pg/ml, the intra-assay variation was 7.8%, while the inter-assay coefficient of variation was 9.3%.

### Daily sperm production per testis, sperm number and transit time in the epididymis

Homogenization-resistant testicular spermatids (stage 19 of spermiogenesis) were counted as described by Robb et al. [41], with adaptations described as follows: the left testes (*n* = 5/group) were decapsulated and homogenized in 500 μL ice-cold saline-triton (0.9% NaCl and 0.05% Triton X-100). After a 5-fold dilution, samples were transferred to Neubauer chambers (4 fields per animal), and mature spermatids were counted. Daily sperm production (DSP) was assessed dividing the number of spermatids at stage 19 by 4.8, which represents the number of days that spermatids are present in the seminiferous epithelium. According to Morais et al. [28], each seminiferous epithelium cycle of *D*. *rotundus* has about 8.23 days and elongated spermatids are present in 58.43% of this cycle. Thus, spermatids are present in the seminiferous epithelium for 4.8 days and this number was used to calculate DSP.

Similar to testis, the caput/corpus and cauda portions of the left epididymides (*n* = 5/group) were homogenized, and sperm were counted as described previously. The transit time of sperm through the epididymis was determined by dividing the number of sperm in each portion of epididymis by the DSP.

### Statistical analysis

Results were analyzed by the Student’s t-test using the GraphPad Prism 6.0 statistical software (GraphPad Software Inc., San Diego, CA, USA), and expressed as mean ± standard error mean (SEM).

## Results

**Fig 1:**
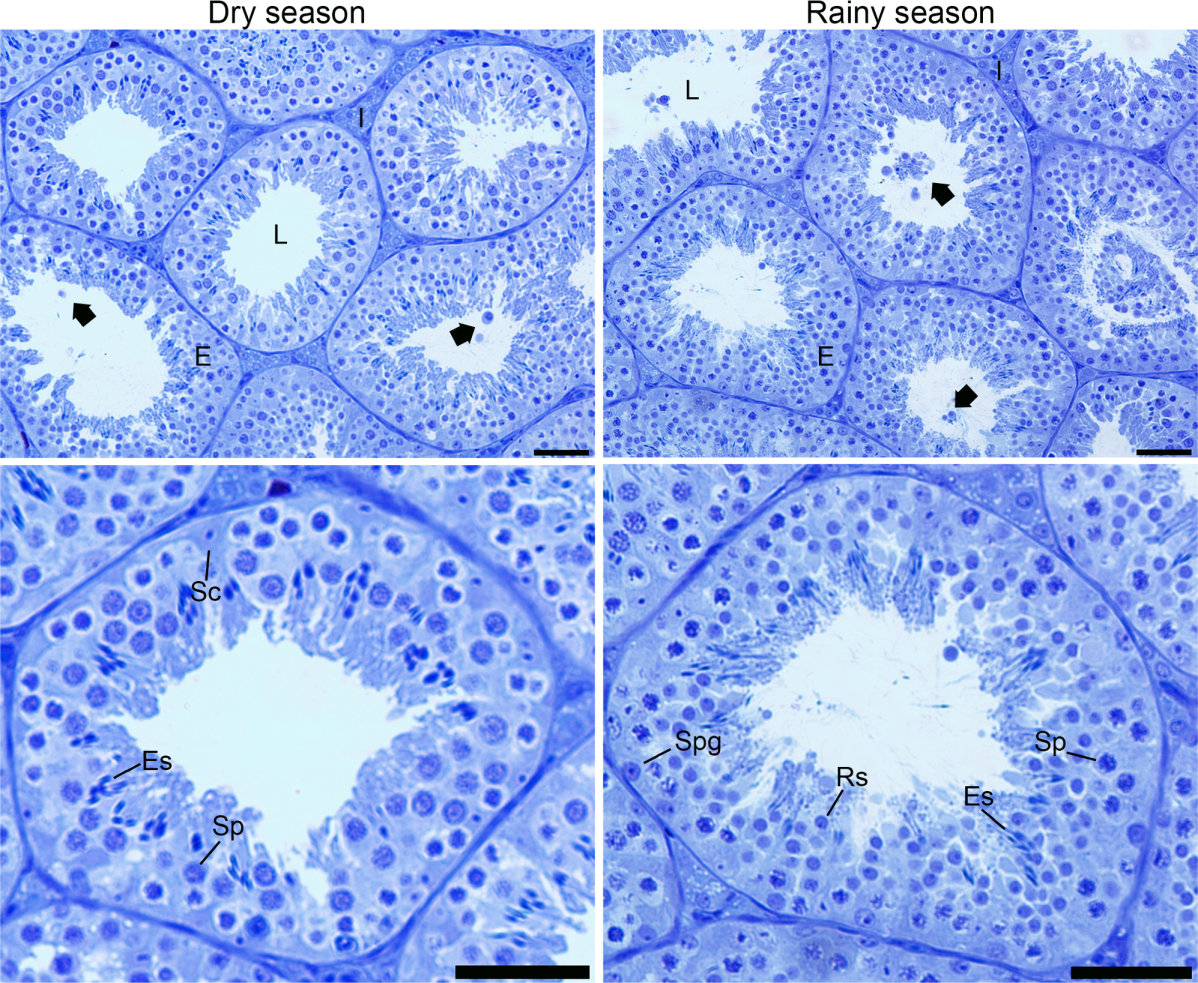
Histological sections from testicular parenchyma of *Desmodus rotundus* in dry and rainy seasons. E = epithelium, L = lumen, I = intertubule, Sc = Sertoli cell, Spg = spermatogonia, Sp = spermatocyte, Rs = round spermatid, Es = elongated spermatid, Black arrow = germ cell. Toluidine blue. Scale bar: 50 μm.

**Fig 2:**
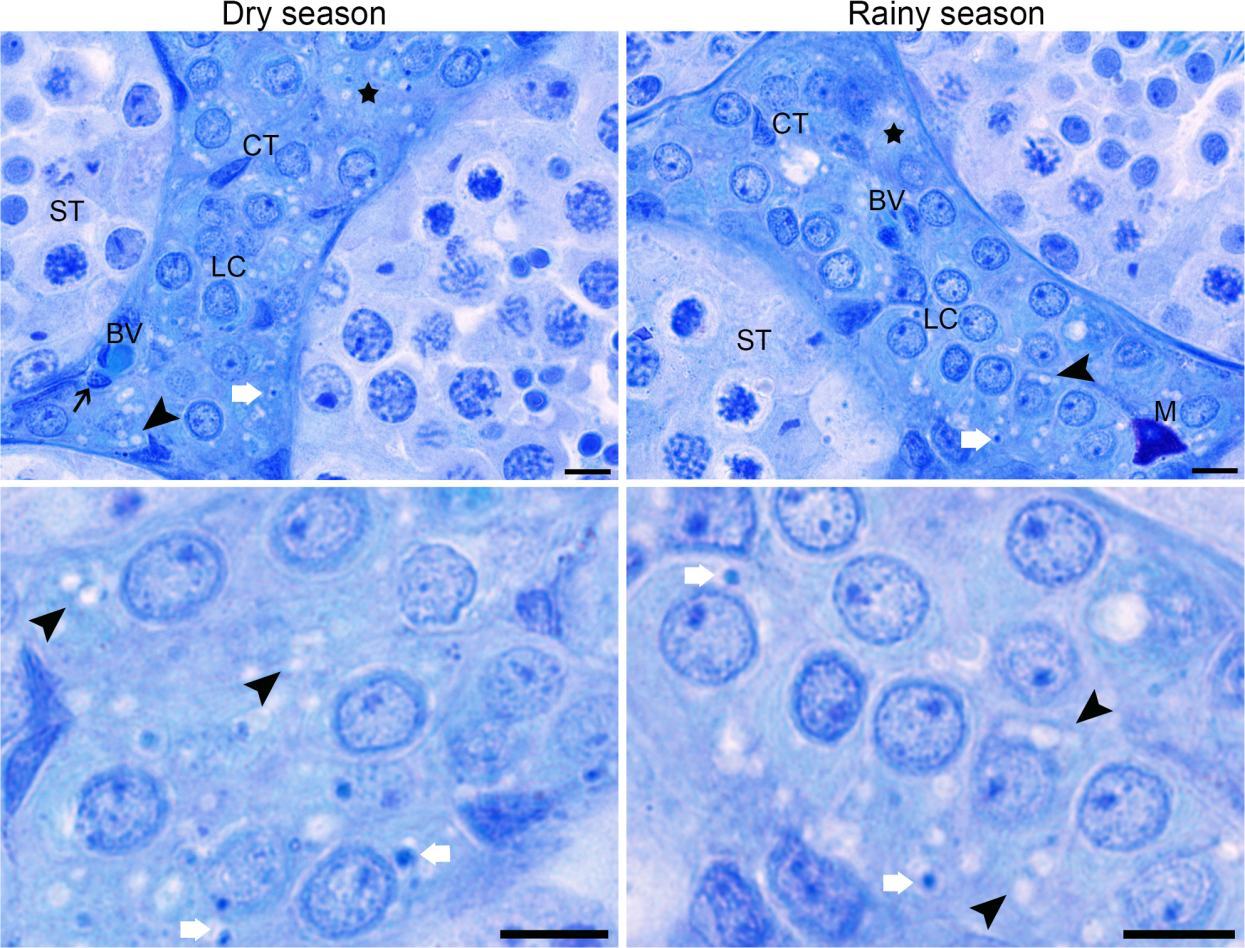
Histological sections from intertubular compartment of *Desmodus rotundus* testis in dry and rainy seasons. ST = seminiferous tubules, LC = Leydig cell, CT = connective tissue, M = mast cell, BV = Blood vessel, White arrow = lipofuscin granules, Arrowhead = lipid droplets, Thin arrow = macrophages. Toluidine blue. Scale bar: 10 μm.

**Fig 3:**
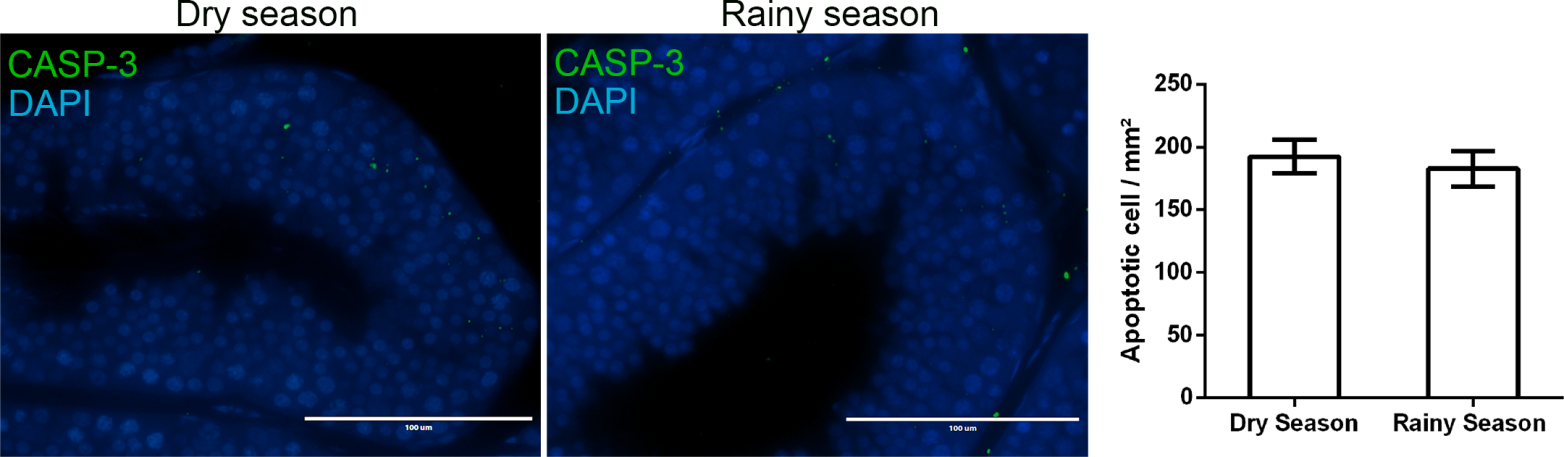
Apoptotic cells in the testis of *Desmodus rotundus* in dry and rainy seasons. Immunolabeling of cleaved casp-3 (green) and DAPI (blue). Scale bar = 100 μm. Mean ± SEM. *P* > 0.05 by Student`s t-test. (*n* = 5/group).

The body and testis weight, as well as the gonadosomatic index did not change in *D*. *rotundus* during dry and rainy seasons (*P >* 0.05; Table 1). Testicular sections of bats in both seasons showed normal tissue architecture, with seminiferous epithelium composed of Sertoli cells and germ cells in different developmental stages (spermatogonia, spermatocytes, round and elongated spermatids), and lumen with spermatozoa (Fig 1). Furthermore, the intertubular compartment was composed of lymphatic space, blood vessels, connective tissue, macrophages, and mainly Leydig cells (Fig 2). It was possible to observe, in both seasons, the presence of germ cells in the lumen of some seminiferous tubules (Fig 1), and lipid droplets and lipofuscin granules in the Leydig cell cytoplasm (Fig 2). Cleaved-caspase 3 labeling was detected in different cell types from both tubular and intertubular compartments of the testis. However, the number of apoptotic cells in the organ did not differ between bats during dry and rainy seasons (*P >* 0.05; Fig 3).

**Table 1:** Biometric and morphometric data of testicular components of *Desmodus rotundus* in dry and rainy seasons.

	Dry season	Rainy season
Body weight (g)	33.28 ± 0.35	33.67 ± 0.44
Testis (g)	0.11 ± 0.01	0.10 ± 0.01
Gonadosomatic index (%)	0.67 ± 0.02	0.59 ± 0.05
Tubular compartment (%)	92.11 ± 0.72	93.07 ± 0.33
Epithelium (%)	78.69 ± 0.45	81.29 ± 0.55**
Tunica propria (%)	3.10 ± 0.26	2.93 ± 0.16
Lumen (%)	10.32 ± 0.30	8.85 ± 0.32*
Intertubular compartment (%)	7.89 ± 0.72	6.93 ± 0.33
Tubular compartment (mL)	0.18 ± 0.01	0.17 ± 0.02
Intertubular compartment (mL)	0.02 ± 0.002	0.01 ± 0.002
Tubulesomatic index (%)	0.54 ± 0.03	0.49 ± 0.05
Tubular diameter (μm)	186.76 ± 2.69	204.24 ± 4.19**
Epithelium height (μm)	65.58 ± 1.45	73.38 ± 1.86*
Tubules total length/testis (m)	6.62 ± 0.30	5.06 ± 0.44*
Tubules total length /g testis (m/g)	29.56 ± 0.66	25.32 ± 0.89**

Mean ± SEM.

* *P <* 0.05 and

** *P <* 0.01 (Student`s t-test) (*n* = 5/group)

The tubular compartment in testes of *D*. *rotundus* in the rainy season presented higher percentage of seminiferous epithelium (*P <* 0.01) and reduced percentage of lumen (*P <* 0.05) compared to animals in the dry season (Table 1). Further, the tubular diameter (*P <* 0.01) and epithelial height (*P <* 0.05) were higher in bat testes during the rainy season in relation to dry season (Table 1). Consequently, the length of seminiferous tubules per testis (*P <* 0.05) and per gram of testis (*P <* 0.01) were lower in bats during the rainy season (Table 1). The other tubular parameters remained unchanged between the groups (*P >* 0.05; Table 1).

Stereological analyses from the testis intertubule showed alteration only in the percentage of connective tissue, which was lower in animals in the rainy season (*P <* 0.05; Table 2). The volume of intertubular components remained unchanged between groups (*P >* 0.05; Table 2). In contrast, morphometric and stereological analyses of Leydig cells showed an increase in nuclear diameter, nuclear percentage, and nuclear volume of these cells in *D*. *rotundus* during the rainy season compared to those in the dry season (*P <* 0.01; Table 3). In addition, the percentage (*P <* 0.01) and volume of cytoplasm (*P <* 0.05) were reduced in these animals (Table 3). The Leydig cell volume, the number of these cells in the testis, and Leydigosomatic index did not differ between the seasons (*P >* 0.05; Table 3).

**Table 2:** Volumetric proportion and volume of the intertubular components in testes of *Desmodus rotundus* in dry and rainy seasons.

	Dry season	Rainy season
*Percentage in the intertubule*		
Connective tissue (%)	6.20 ± 0.27	5.44 ± 0.07*
Lymphatic space (%)	6.76 ± 0.24	7.28 ± 0.26
Blood vessel (%)	13.96 ± 0.26	14.88 ± 0.40
Macrophages (%)	0.64 ± 0.13	0.86 ± 0.16
Leydig cell (%)	72.44 ± 0.25	71.54 ± 0.57
*Volume per testis*		
Connective tissue (μL)	0.94 ± 0.08	0.68 ± 0.01
Lymphatic space (μL)	1.06 ± 0.16	0.92 ± 0.15
Blood vessel (μL)	2.18 ± 0.31	1.90 ± 0.31
Macrophages (μL)	0.10 ± 0.02	0.11 ± 0.02
Leydig cell (μL)	11.25 ± 1.44	9.00 ± 1.28

Mean ± SEM.

* *P <* 0.05 (Student`s t-test) (*n* = 5/group)

**Table 3:** Leydig cell morphometry and stereology of *Desmodus rotundus* in dry and rainy seasons.

	Dry season	Rainy season
Nuclear diameter (μm)	6.65 ± 0.05	6.96 ± 0.06**
Nuclear percentage (%)	17.50 ± 0.50	22.22 ± 0.80**
Cytoplasm percentage (%)	82.50 ± 0.50	77.78 ± 0.80**
Nucleus volume (μm^3^)	154.18 ± 3.59	176.56 ± 4.47**
Cytoplasm volume (μm^3^)	729.08 ± 28.04	591.20 ± 33.06*
Leydig cell volume (μm^3^)	883.25 ± 29.76	800.78 ± 45.68
Number of Leydig cells/testis (x10^6^)	6.34 ± 0.74	5.72 ± 0.90
Number of Leydig cells/g testis (x10^6^)	56.38 ± 5.73	55.91 ± 5.03
Leydigosomatic index (%)	0.034 ± 0.004	0.027 ± 0.004

Mean ± SEM.

* *P <* 0.05 and

** *P <* 0.01 (Student`s t-test) (*n* = 5/group)

**Fig 4:**
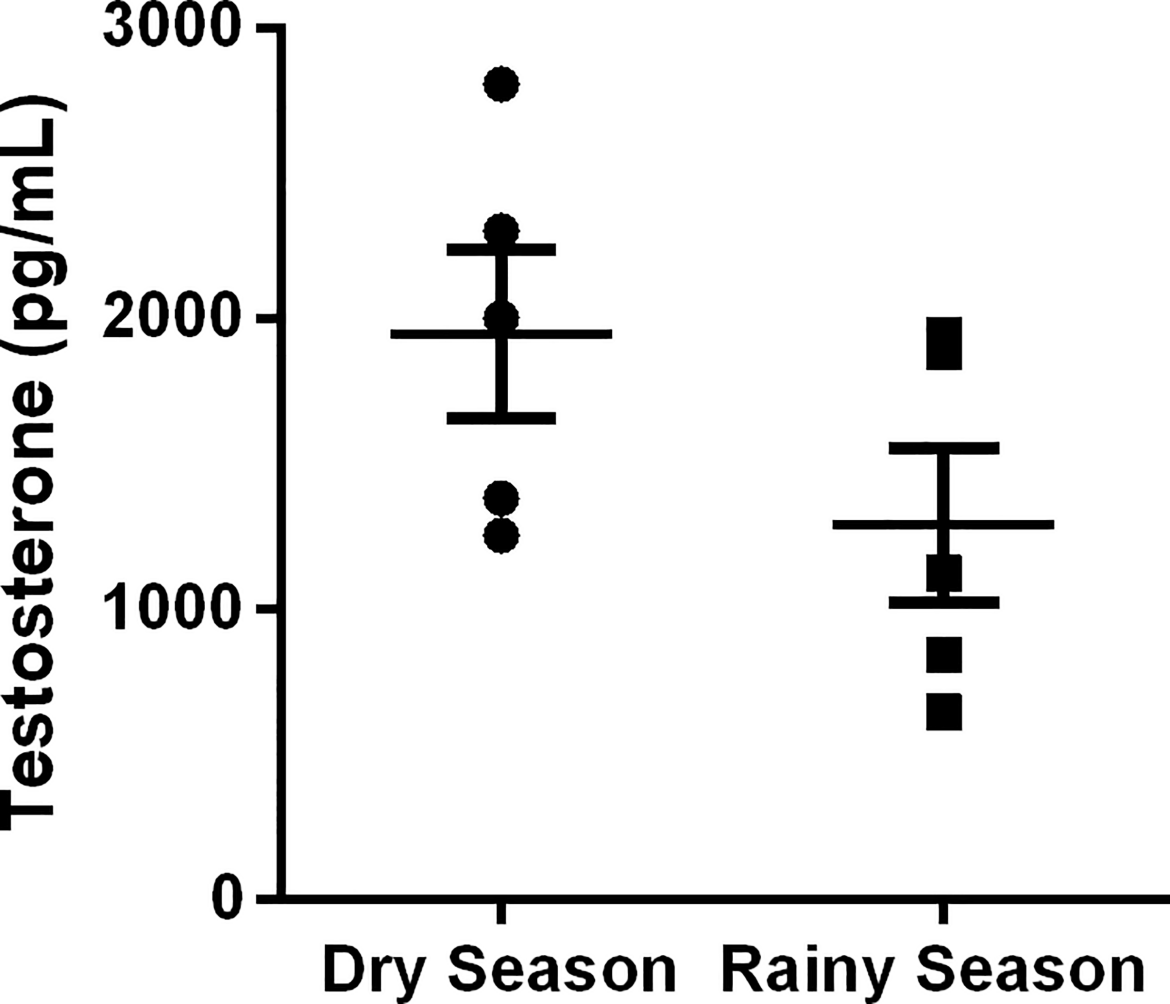
Serum testosterone concentration of *Desmodus rotundus* in dry and rainy seasons. Mean ± SEM. *P* > 0.05 by Student`s t-test. (*n* = 5/group).

The concentration of serum testosterone also did not change between the animals in dry and rainy seasons (*P >* 0.05; Fig 4). However, in the rainy season, *D*. *rotundus* presented the highest number of spermatid per gram of testis (*P <* 0.01) and sperm in caput/corpus epididymis and per gram of caput/corpus (*P <* 0.05; Table 4). Inversely, they presented the lowest number of sperm in the cauda epididymis (*P <* 0.05), as well as per gram of cauda (*P <* 0.01; Table 4). In addition, the sperm transit time in the cauda epididymis was reduced in these animals when compared to bats in the dry season (*P <* 0.01, Table 4). The spermatid number per testis, daily sperm production and sperm transit time in caput/corpus epididymis did not change between the seasons (*P >* 0.05; Table 4).

**Table 4:** Sperm count parameters in the testis and epididymis of *Desmodus rotundus* in dry and rainy seasons.

	Dry season	Rainy season
Spermatid number (x10^6^/testis)	4.75 ± 0.25	5.27 ± 0.32
Spermatid number (x10^6^/g testis)	61.69 ± 5.57	89.00 ± 4.99**
Daily sperm production (x10^6^/testis/day)	1.04 ± 0.03	1.09 ± 0.07
Caput/corpus epididymis sperm number (x10^6^/organ)	0.93 ± 0.08	1.46 ± 0.15*
Caput/corpus epididymis sperm number (x10^6^/g organ)	99.63 ± 5.99	117.2 ± 3.06*
Sperm transit time in the caput/corpus epididymis (days)	1.03 ± 0.06	1.18 ± 0.12
Cauda epididymis sperm number (x10^6^/organ)	2.08 ± 0.29	1.21 ± 0.18*
Cauda epididymis sperm number (x10^6^/g organ)	171.1 ± 13.39	111.88 ± 6.67**
Sperm transit time in the cauda epididymis (days)	2.08 ± 0.24	1.08 ± 0.12**

Mean ± SEM.

* *P <* 0.05 and

** *P <* 0.01 (Student`s t-test) (*n* = 5/group)

## Discussion

The present study compared male reproductive parameters of the common vampire bat *D*. *rotundus* during dry and rainy seasons. To our knowledge, this is the first study to evaluate testicular morphology, testosterone production, and epididymal function associated with seasons in this species. Our results showed that stereological parameters of the testis changed in the rainy season, as well as sperm counts and sperm transit time in the epididymis.

Overall, the histology of testicular parenchyma in *D*. *rotundus* observed herein was similar to that previously described in this species by Morais et al. [28]. Likewise, no differences in morphological parameters between dry and rainy seasons were observed in testis of *Sturnira lilium*, another phyllostomid species widely distributed in Brazil [7]. It is known that several bat species, including *D*. *rotundus*, did not present hibernation and testis regression [3]. This fact supports our findings regarding maintenance of testis tissue architecture in dry and rainy seasons, as well as the body and testis weight and gonadosomatic index. Previously, alterations in testis weight and histomorphometry throughout the year, as well as the histology of seminiferous tubules were described in *Artibeus lituratus*, *Myotis nigricans* and *Myotis levis* [6,42,43]. In these cases, atrophy of seminiferous tubules with presence of Sertoli cells and spermatogonia were described in regressing testes, in contrast to the histology showing full spermatogenesis in testes during the reproductive period [6,43]. In the current study, the only histological alteration observed in *D*. *rotundus* was the presence of detached germ cells in the lumen of seminiferous tubules, which might have been a consequence of the constant number of apoptotic germ cells found in the tubule. Taboga et al. [44] also observed detachment of germ cells in other phyllostomids and concluded that these cells undergo spontaneous cell death by apoptosis in the testis and follow to be deposited in the epididymis. Collectively, our results confirm that *D*. *rotundus* did not present regression period, differently to other bat species captured in Brazil [6,42,43].

Despite the maintenance of testicular morphology in both seasons, stereological analyses showed variations in testis components in the rainy season. The increase in the percentage of epithelium was probably compensated by the decrease in lumen percentage, maintaining the proportion of tubules and the testicular tissue architecture of bats. In addition, the percentage of epithelium might have been influenced by the increased epithelial height that, in turn, was responsible for the greater tubular diameter observed herein. Such finding was probably reflected in a decrease of seminiferous tubules length also found in animals from the rainy season. Notably, seminiferous epithelium is the testicular component directly related to spermatogenic activity [45]. Therefore, the measurement of the epithelial height is used to evaluate sperm production, and it indicates the investment in spermatogenesis [14]. Based on that, we may suggest that, in the rainy season, the seminiferous epithelium presented an intense activity to produce mature cells after the mating season in order to prepare the male to the next reproductive season. In fact, the rainy season is considered the preferred season for births and lactation by female *D*. *rotundus* [26,27]. Consequently, it is likely that mating occurs between the end of autumn and early winter (dry season), since the gestation period is about seven months [27].

The increased number of sperm in caput/corpus regions and its reduction in the epididymis cauda observed in the rainy season might also be a consequence of the reproductive behavior observed in these bats. As sperm are constantly produced and released [46], it is possible that spermatozoa from testis followed to the epididymis increasing their quantity in proximal regions of the organ. Meanwhile, the low sperm number in cauda epididymis indicates that sperm reserves were used for mating during the end of dry season. It was then reflected in the reduced sperm transit time observed herein. It is known that the number of sperm in each epididymal region relies on the sperm transit time, which is a process highly regulated by androgens [47]. As some captures were performed at the beginning of each season, probably the sperm reserves in epididymis have not returned to normal yet.

Moreover, alterations in morphometric parameters of the intertubule have been reported in testes of other bat species during the rainy season [13,48]. Herein, the percentage of connective tissue and Leydig cell structure changed in the intertubule of *D*. *rotundus* during the rainy season, with no damages to the structural organization of the intertubular compartment. Specifically, alterations observed in stereological parameters of Leydig cells (nuclear diameter, proportion and volume of nucleus and cytoplasm) might have contributed for the maintenance of their volume and number in both dry and rainy seasons, with no influences on testosterone levels. Typically, Leydig cells are considered the endocrine portion of the testis being responsible for the production of testosterone, necessary for the development and maintenance of secondary sex characteristics, as well as the maintenance of spermatogenesis [14]. In both seasons, testosterone production in Leydig cells may have been confirmed by the presence of lipid droplets and lipofuscin granules in their cytoplasm, besides the results of serum testosterone. While the former indicates the presence of precursors for androgen biosynthesis within this cell type, the latter represents the presence of semidegraded form of lipids used for hormonal production [16, 49]. Some authors showed variation in serum testosterone concentration associated with morphological changes in Leydig cells in seasonal bats [50,51]. In these animals, Leydig cells were described as hypertrophic cells during the reproductive season, while during the testicular regression period the cell size and the number of lipofuscin granules were significantly reduced [50,51]. It is known that animals with polygamous behavior such as bats have great percentage of Leydig cells in the intertubule and, consequently, greater androgenic capacity when compared to monogamous species [48,52,53]. Thus, the largest percentage of Leydig cells observed in the intertubule of *D*. *rotundus* in both seasons is in accordance with its social behavior, and the stable number of these cells can explain the testosterone levels observed in dry and rainy seasons. Altogether, these findings indicate that morphological parameters underpin the results of testosterone production found in *D*. *rotundus*. Further, the acclimatization period used here was enough to minimize physiological changes caused by capture [34,35].

Finally, testosterone levels sustained the number of spermatids and the daily sperm production in dry and rainy seasons. Interestingly, our data of daily sperm production in *D*. *rotundus* were lower compared to the findings reported by Morais et al. [28]. This probably occurred because the methodology adopted in the present study considers only the number of testicular homogenized-resistance spermatids heads (HRSH) in order to calculate sperm production. The HRSH are considered viable cells, and they would probably become sperm in the end of spermatogenic cycle [54]. Conversely, the methodology used by Morais et al. [28] is based on the round spermatids count in histological testis sections in stage 1 of the seminiferous cycle, regardless of cell loss. There is no other reason that could explain the differences observed for daily sperm production in both techniques.

In the light of the foregoing, Fleming et al. [55] proposed that the reproductive cycles of tropical bat species are directly influenced by the viability of food supply and climatic factors (e.g., temperature, rainfall, photoperiod). For instance, in insectivorous bats, these cycles may be influenced by abiotic factors only in an indirect way by variation in food resources [6]. Herein, we may suggest that these factors do not influence the male reproductive pattern in *D*. *rotundus*. Their hematophagous feeding habits, as well as their habit to inhabit caves and abandoned buildings next to livestock promote year-round abundant feeding resources [18,19]. Notably, while in the rainy season we have sampled in October, in the dry season the specimens were collected in April and June. We acknowledge that climate variations may occur between the dry season´s months. Nevertheless, no alterations were observed in the main parameters described herein. Additionally, it is likely that females of *D*. *rotundus* are not influenced by photoperiod being considered polyestrous [3,27]. Of note, this fact strongly influences the male reproductive behavior [1].

## Conclusion

Taken together, we can conclude that stereological and morphometric parameters in the testis of the common vampire bat *D*. *rotundus* varied between dry and rainy seasons. However, the maintenance of testosterone concentration and daily sperm production indicates that the reproductive pattern in *D*. *rotundus* does not change between seasons. Studies involving sampling in every month throughout the year, and molecular approaches will be very important to confirm the reproductive pattern here presented. Notwithstanding, our data can be a baseline for management of vampire bat population as an attempt to control rabies disease.
